# Typhoid fever in Santiago, Chile: Insights from a mathematical model utilizing venerable archived data from a successful disease control program

**DOI:** 10.1371/journal.pntd.0006759

**Published:** 2018-09-06

**Authors:** Jillian S. Gauld, Hao Hu, Daniel J. Klein, Myron M. Levine

**Affiliations:** 1 Institute for Disease Modeling, Bellevue, Washington, United States of America; 2 Center for Vaccine Development and Global Health, University of Maryland School of Medicine, Baltimore, Maryland, United States of America; Johns Hopkins Bloomberg School of Public Health, UNITED STATES

## Abstract

Typhoid fever is endemic in many developing countries. In the early 20^th^ century, newly industrializing countries including the United States successfully controlled typhoid as water treatment (chlorination/sand filtration) and improved sanitation became widespread. Enigmatically, typhoid remained endemic through the 1980s in Santiago, Chile, despite potable municipal water and widespread household sanitation. Data were collected across multiple stages of endemicity and control in Santiago, offering a unique resource for gaining insight into drivers of transmission in modern settings. We developed an individual-based mathematical model of typhoid transmission, with model components including distinctions between long-cycle and short-cycle transmission routes. Data used to fit the model included the prevalence of chronic carriers, seasonality, longitudinal incidence, and age-specific distributions of typhoid infection and disease. Our model captured the dynamics seen in Santiago across endemicity, vaccination, and environmental control. Both vaccination and diminished exposure to seasonal amplified long-cycle transmission contributed to the observed declines in typhoid incidence, with the vaccine estimated to elicit herd effects. Vaccines are important tools for controlling endemic typhoid, with even limited coverage eliciting herd effects in this setting. Removing the vehicles responsible for amplified long-cycle transmission and assessing the role of chronic carriers in endemic settings are additional key elements in designing programs to achieve accelerated control of endemic typhoid.

## Author summary

Typhoid fever was successfully controlled in Santiago, Chile, after a series of interventions including vaccination with a live oral vaccine (Ty21a), and an environmental sanitation improvement, when a ban was put on the irrigation of salad vegetable crops with untreated sewage. Data collected during this period inform seasonality, age distribution and longitudinal trends of disease. We developed an individual-based, mathematical model to both simulate the dynamics of typhoid seen in Santiago, as well as to investigate relative impacts of the vaccine and sanitation interventions. We found that herd immunity resulted from field trials of the Ty21a vaccine and that chronic carriers were a likely driver of sustained transmission at low incidence levels. Modeling typhoid fever in areas that have demonstrated successful control provides insight for control strategies in modern settings.

## Introduction

Typhoid fever caused by *Salmonella* Typhi was controlled in developed countries after widespread water and sanitation improvements were introduced, but remains a pressing public health problem in many developing countries [1–3], with estimates of global burden ranging from approximately 10 to 20 million cases per year [4–7]. Effective control of typhoid is often impeded by lack of knowledge of the local dominant transmission routes, age-specific incidence of disease and role played by chronic carriers (individuals with *S*. Typhi-colonized gallbladders who can transmit the pathogen for decades). Modeling endemic typhoid in specific epidemiologic niches can guide investments and prioritization of control strategies such as identifying cost-effective targets for immunization with new and existing typhoid vaccines and improving water/sanitation/hygiene (WASH) infrastructure [8–11]. The usefulness of mathematical models is enhanced when comprehensive and precise input data are available. Regrettably, such data are often unavailable where typhoid is currently endemic. Thus, modeling data from sites where typhoid has already been successfully controlled offers an opportunity to dissect the mechanisms of transmission in those data-rich settings and also allows those models to be adapted to study transmission and the impact of potential interventions in modern endemic settings.

Enigmatically, typhoid was hyper-endemic in Santiago, Chile from the mid-1970s through early 1990s [12], even though 96% of Santiago households had access to treated, bacteriologically-monitored water and ~80% had toilets connected to the municipal sewerage system [12–14]. Typhoid fever incidence in Santiago was stable through the early 1970s but doubled in 1977 and 1978 without an obvious explanation [12,13]. This prompted the Head of the Epidemiology Unit of the Ministry of Health of Chile (MoHC), Dr. José-Manuel Borgoño, and the Pan American Health Organization in 1978 to invite two external advisers to Chile, Drs. Branco Cvjetanovic and Myron M. Levine, to provide independent unbiased assessments of the endemicity of typhoid and offer recommendations. One recommendation was to establish a Typhoid Fever Control Program (TFCP), which was instituted in 1979 by Dr. Borgoño with Dr. Levine as external adviser.

During the ensuing 13 years, the Chilean TFCP: strengthened clinical, epidemiologic and bacteriological surveillance within the Metropolitan Region (Santiago and environs) [13]; quantified the reservoir of chronic gallbladder typhoid carriers (prevalence, 694/10^5^ adults) [15,16]; identified risk and protective factors for transmission [14]; hypothesized that an unusual mechanism of amplified transmission was maintaining hyper-endemic typhoid disease across all socioeconomic levels and neighborhoods in an urban population with widespread access to potable water and flush toilet sanitation [17,18]; and undertook environmental bacteriology investigations to confirm the hypothesis [17,18]. The environmental bacteriology studies identified irrigation of crops with untreated raw sewage wastewater during the rainless summer months as the predominant mechanism that was sustaining amplified transmission [17,18]; 90% of the crops were vegetables (lettuce, cabbage, celery) eaten uncooked. A computer-based model of endemic typhoid in Santiago was developed based on pre-intervention incidence data (1968–1976) to explore the impact of future vaccination and sanitation interventions to control typhoid [8].

Two major preventive interventions were instituted in Santiago during the period of the TFCP, with each followed by marked drops in typhoid incidence. First, from 1982 through 1991, were four large-scale field trials of Ty21a live oral vaccine among 514,150 schoolchildren, the age group that accounted for >60% of cases of typhoid [19–24]. Second was a sanitation intervention. In April 1991, following an outbreak of 41 confirmed cholera cases that occurred in Santiago [25], the practice of irrigating crops with raw sewage-containing wastewater was prohibited [13,25]. Thenceforth, this strictly-enforced intervention abruptly interrupted the long-standing amplified transmission of typhoid in Santiago [13,18].

The availability of data from an extended period of endemic transmission and well-documented non-coincident vaccine and sanitation interventions with surveillance across both time periods offered unique data to examine the drivers of endemicity in Santiago, and to estimate the impacts of multiple interventions across a single population. By examining assumptions and constraints through mathematical modeling of data from a historical site, one can better understand typhoid transmission in current hyper-endemic loci.

## Methods

### Model structure

The typhoid model was built upon the EMOD 2.11 framework [26]. The structure was created by modifying a previous individual-based typhoid model [27]. Modifications include adding multiple transmission routes and simplifying immunity; sterilizing and clinical immunities were combined into a single immunity structure, absent data to inform individual durations. Santiago’s population was simulated using age-specific fertility and mortality rates estimated with Instituto Nacional de Estadisticas census data [28]. The model was initialized with the earliest reported age distribution, with individuals entering and exiting the model through age-specific fertility and mortality.

Typhoid transmission occurs through either the “short-cycle” or “long-cycle”. Short-cycle denotes infections transmitted from person-to-person through proximate contaminated food vehicles [29]. Long-cycle signifies infections transmitted through environmental mediators such as contaminated water [30], crops irrigated with untreated sewage [18] or widely-distributed contaminated commercial food products [31]. Our model captures both transmission routes; individuals can both contaminate and become exposed to the short-cycle and long-cycle “composite of contaminated vehicles of transmission,” or CCVT.

Infectious individuals shed into both short-cycle and long-cycle CCVTs at a daily rate per their infectiousness, in colony-forming units (CFU). In the absence of quantitative shedding data, this value is an estimated free parameter for acute infectiousness (AI, Table 1), with multipliers for non-acute disease states, explained in detail below. The die-off of *S*. Typhi over time outside the human body varies in different environmental niches [32,33]. Our estimate of the long-cycle CCVT daily decay at rate LD (Table 2) was based on the *S*. Typhi die-off results reported by Cho et al [32]. Assuming short-cycle transmission ensues primarily via proximate food vehicles that are prepared and consumed daily, short-cycle CCVT decays at 100% each day.

**Table 1:** Fitted parameters in model.

Parameter	Abbreviation	Range	Fitted value
Acute infectiousness (CFU)	AI	0–100000	13436
Reduction in susceptibility after clinical or sub-clinical infection	P	0–1	0.998
Long-cycle exposure seasonality	EPS	1–365	Peak start day of year: 290
	PD		Peak duration (days): 108
	RDD		Ramp down duration (days): 227
	RUD		Ramp up duration (days): 11
Daily number of short-cycle exposures*	ES	~Pois(0–1)	~Pois(0.009)
Daily number of long-cycle exposures*	EL	~Pois(0–1)	~Pois(0.375)
Chronic carrier probability given gallstones	pC	0–1	0.108
Probability of infection being symptomatic	pA	0–1	0.053
Chronic infectiousness (relative to acute)	rC	0–1	0.241
Susceptible introduction curve	S	-1–50	0.925
Long-cycle exposure multiplier	mEL_A	0–1	0.541
	mEL_B		0.323
	mEL_C†		[0.226, 0.529]
Ty21a vaccine duration of efficacy (days)	D	1000–3650	3063

Parameters marked with * are Poisson distributions ~Pois(λ) with fitted rate λ. Parameters marked with † indicate linear interpolation between fitted values.

**Table 2:** Assumed and literature derived parameters.

Parameter	Symbol	Value	Source
Incubatory infectiousness, relative to acute illness	rI	0.5	Assumption [36,37]
Subclinical infectiousness, relative to acute clinical illness	rS	1	Assumption
Incubation duration (days)*	PD	Low: Lognormal (2.235, 0.496)	[38]
		High: Lognormal (1.548, 0.344)	
Duration of infectiousness (weeks)*	ID	Under 30 years: Lognormal (1.172, 0.483)	[39]
		30 years old +: Lognormal (1.258, 0.788)	
Probability of gallstones by age bin (male, female)	pG	10–19 (0, 0.097)	[15,16]
		20–29 (0.045, 0.234)	
		30–39 (0.134, 0.431)	
		40–49 (0.167, 0.517)	
		50–59 (0.198, 0.60)	
		60–69 (0.247, 0.692)	
		70–79 (0.435, 0.692)	
		80+ (0.40, 0.555)	
Long-cycle environmental vehicle decay rate	LD	0.24/day	[32]
Dose response†		beta-Poisson(1.75E-01,1.11E+06)	[40]
Long-cycle exposure multiplier, 1992–2000	mEL_D	0	Assumption

Parameters marked with * are lognormal distributions Lognormal(μ,σ), where μ and σ are the fitted mean and standard deviation values of the underlying normal distribution, respectively. Parameter marked with † is a beta-Poisson model parameterized beta-Poisson(α, N_50_), where α and N_50_ are derived from published sources [40].

The number of times a susceptible individual is exposed to each CCVT is determined by a daily Poisson process, with rates for both short-cycle and long-cycle (EL, ES, Table 1) left free for model fitting. In the model, the probability of clinical response post-exposure differs by transmission route. Short-cycle transmission is assumed to always involve a food vehicle that protects *S*. Typhi against gastric acid or contains a large inoculum (or both). Thus, we assume that small inocula that may not transmit through the long-cycle are successfully transmitted by short-cycle vehicles. Thus, short-cycle is modeled in a direct transmission framework, where probability of infection is the population-scaled short-cycle CCVT divided by the total potential short-cycle CCVT.

We undertook to address the heterogeneity inherent in long-cycle transmission, which leads to potential variation in inoculum size (i.e., water-borne vs. food-borne long-cycle exposure). Therefore, the probability of infection through the long-cycle is determined by a dose-response function where population-scaled CCVT for the long-cycle is the infecting inoculum size. The dose-response function is a beta-Poisson curve fitted to Maryland experimental challenge dose-response data where *S*. Typhi inocula were administered without buffer (Table 2) [34,35]. We assume Maryland challenge data represent Santiago as a whole, absent additional data.

**Fig 1:**
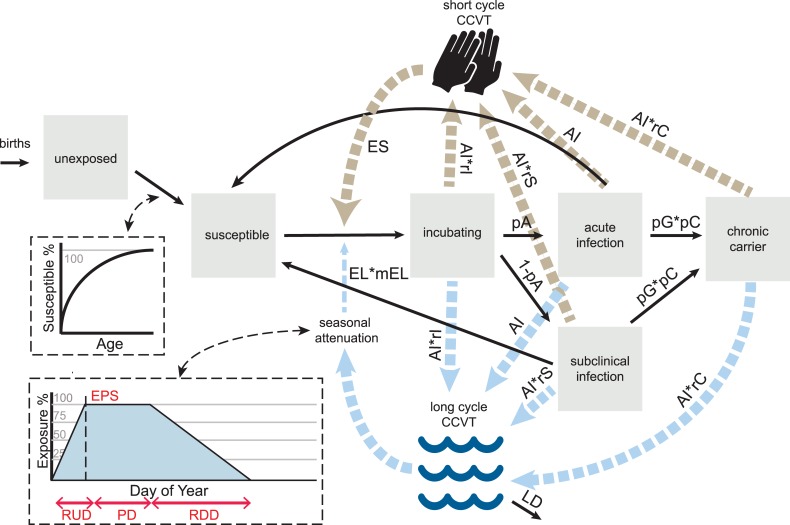
Typhoid model framework. Transmission route diagram for typhoid model with literature-derived and fitted parameters, described in Tables 1 and 2. Disease state-specific contributions to short- and long-cycle composite of contaminated vehicles of transmission (CCVT) are represented, along with seasonal attenuation and age-susceptibility mechanisms.

Infected individuals in the model transition through disease states beginning with the incubation period (Fig 1), an asymptomatic infectious period monitored via stool cultures in typhoid challenges [36,37]. The incubation period is informed by the Maryland challenge model [38], which demonstrated shorter incubations for those challenged with high (10^8^−10^9^ CFU) versus lower (10^5^ CFU) inocula. Incubation period in the model can be drawn from one of two estimated lognormal distributions [38], with the cutoff for high vs. low dose being the mid-point between 10^5^ and 10^8^ CFU.

We are unaware of direct comparisons of *S*. Typhi counts in stools of incubating, sub-clinical and clinical infections. To approximate, we used isolation rates from stool cultures to inform the relative infectiousness of the incubation period versus acute clinical infections. In the Oxford human challenge model, 26% of stools cultured within 72 hours post-challenge were positive for *S*. Typhi among those eventually diagnosed with typhoid disease [37]. Similarly, the maximum percentage of positive stools in the first three days post-challenge was ~30% in the Maryland model, assuming equal stool isolation rates each day [36]. In the following week, the maximum daily percentage of positive stool cultures was approximately 60%. The pre-antibiotic era report of Ames and Robins described stool isolation rates to be in the range of 60–70%, during the three weeks following diagnosis of clinical illness [39]. Using these isolation rates as a proxy for relative infectiousness, we assume a baseline relative infectiousness, rI, of 0.5 for incubating individuals compared to acute cases (Table 2).

Individuals who excrete virulent *S*. Typhi following ingestion of an inoculum may or may not develop clinical typhoid [34], and clinical severity does not correlate with infectious dose [34,37,38,41]. Individuals progress to either clinical or subclinical infection, according to probability of clinical infection pA. During the primary bacteremia of acute typhoid infection, be it clinical or subclinical, *S*. Typhi always reaches the gallbladder [42]. Further, older adults have abnormal gallbladder mucosa more often than younger adults or children. Therefore, even though all adults with gallbladder disease who have acute typhoid fever or acute sub-clinical infection do not become chronic carriers, they may nevertheless have delayed clearance of *S*. Typhi from their gallbladder. This feature was observed by Ames and Robins in the pre-antibiotic era [39]. Duration of clinical and subclinical shedding is sampled from a lognormal distribution, stratified by <30 and ≥30 years of age, derived from non-chronic carrier shedding durations of acute infections [39].

Following the infectious period, individuals can revert to susceptible class or become chronic carriers. Both clinical and subclinical infections may lead to chronic carriers in this model, and the probability of becoming a carrier after each infection is age- and gender-specific. The propensity for *S*. Typhi (or *S*. Paratyphi A or B) to reside long-term in the gallbladder is related to whether a patient has chronic gallbladder disease due to gallstones [43,44], though long-term carriage may occasionally occur in persons without gallstones. Regardless, the prevalence of chronic typhoid carriers parallels the prevalence of cholelithiasis and chronic gallbladder disease. Both are much greater in females than males and the prevalence increases with age [15]. Our model utilizes the sex- and age-specific prevalence of gallstones in Santiago [15], and multiplies this value by an additional free parameter, pC, to inform the probability of chronic carriage per infection when an individual has gallstones.

**Fig 2:**
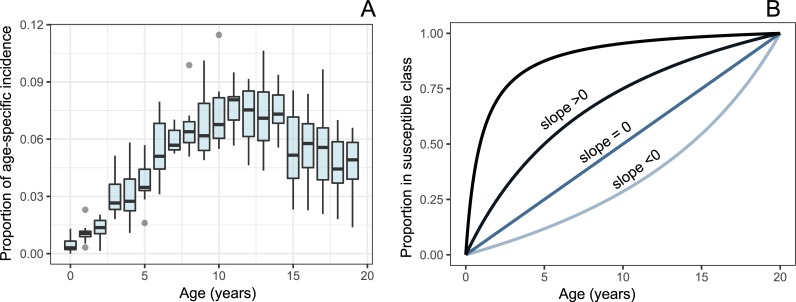
Age distributions of typhoid in Santiago and the model. A. Age-specific incidence in Area Norte, Santiago (by year of age) B. Age-specific exposure mechanism with equation 1-(20- A) / (A*S + 20), where A is age in years, and S is the slope of the curve.

When examining the age-specific incidence of typhoid fever in Area Norte, Santiago, from 1971–1981, there appear to be abrupt increases in incidence at 3 and 6 years of age (Fig 2A). As these are the common entry ages of preschool and elementary school, this finding suggests that differential age-specific exposures may influence the occurrence of pediatric typhoid. Our model assumes all individuals are born into an unexposed class and move to the susceptible class at probabilities for each age. Specifically, at each month of age a fitted curve determines the probability of an individual entering the susceptible class. The curve is anchored at 0% exposure at birth, and 100% exposure at age 20 years, with a free slope parameter (S) determining the concavity/shape of the function (Fig 2B).

We include a mechanism for reinfection with immunity in our model. All individuals enter the model with no prior infections (Ni = 0). When an individual returns to the susceptible class after a subclinical or clinical infection, the number of previous infections, Ni, increases by 1. Upon exposure to either short-cycle or long-cycle CCVT, the probability of becoming infected is multiplied by the value resulting from the equation: (1-P)^Ni^. Currently, there is no decay of Ni over an individual’s life because we assume that in the hyper-endemic Santiago setting, repetitive exposures to *S*. Typhi achieve immunologic boosting. This assumption is reasonable for endemic settings with frequent exposures to *S*. Typhi but may not hold where typhoid endemicity is unstable or when individuals leave the hyper-endemic setting. P, the reduction in susceptibility after clinical or sub-clinical infection, is left as a free parameter to be fitted to Santiago dynamics.

An individual’s dose through long-cycle transmission in the model is attenuated by a mechanism for seasonality, chosen to represent the likely constant shedding of individuals into the long-cycle vehicle but with differential exposure to it influenced by seasonal crop selection and need for irrigation. In the high (warm, rainless) season, we assume individuals are exposed to 100% of long-cycle CCVT. At low season, we assume no exposure to long-cycle CCVT. Degrees of exposure in the intermediate stages are mediated by a trapezoidal function, where ramp-up and ramp-down durations (RUD and RDD), in combination with high-season duration and timing (PD, EPS), determine a linear function connecting high and low seasons outlined in Fig 1. These parameters are left free for model fitting.

Four large-scale field trials of Ty21a vaccine encompassing 514,150 schoolchildren were performed in Santiago, beginning in 1982 [19–24]; three trials included placebo control groups. The timing and number of vaccinees for each of the trial years are summarized in Table 3, with age ranges restricted between 6 and 17 years of age in the absence of exact age distributions. Formulation, number of doses and inter-dose intervals varied in the trials, affecting vaccine efficacy and duration of protection (Table 3).

**Table 3:** Summary of results of Ty21a vaccine field trial in Santiago, Chile.

Date of vaccination	Formulation	Doses	Efficacy	Evaluated/effective years	Age range in years	Number	% coverage metropolitan Santiago age group	Reference
May 26-June 2, 1982	Enteric coated capsule	2	59%	2	6–9	8,010	0.022	Black et al 1990 [20]
					10–14	11,048	0.026	
					15–17	8,562	0.033	
		1	29%	2	6–9	8,009	0.022	
					10–14	11,047	0.026	
					15–17	8,562	0.033	
Mid-July-early September 1983	Enteric coated capsule, short interval	3	62%†	7†	6–17	22,168	0.021	Levine et al 1987 [19]
	Enteric coated capsules, long interval	3	49%	3	6–17	21,598	0.022	
	Gelatin capsules, long interval	3	31%	3	6–17	21,541	0.020	
	Gelatin capsules, short interval	3	19%	3	6–17	22,379	0.021	
October 3–22, 1984	Enteric coated capsule	2	59%*	2*	6–17	66,615	0.074	Ferreccio et al 1989 [21]
		3	62%†	7†	6–17	64,783	0.073	
		4	62%†	7†	6–17	58,421	0.065	
September-October 1986	Enteric coated capsules	3	62%†	7†	6–9	21,128	0.069	Levine et al 1990 [22]
		3	62%†	7†	10–17	13,568	0.022	
	Suspension of reconstituted lyophilate in liquid buffer	3	78%†	5†	6–9	22,586	0.073	
		3	78%†	5†	10–17	14,037	0.023	

Parameters marked with * are derived from Black et al [20] * and those marked with † from Levine et al 1999 [23] and not from the trial year outcomes. Highlighted trials are categorized as doses equivalent to full efficacy doses or better.

Vaccination is implemented as a multiplier on an individual’s probability of infection, equal to one minus the vaccine efficacy corresponding to the year and dose listed in Table 3. The multiplier exists for the duration listed for partially protective formulations (Table 3), or the estimated parameter, D, for trials equivalent to full efficacy doses or better (Table 1). After the defined duration of protection, there is no residual immunity or protection assumed in the model, and individuals return to a fully susceptible state. This choice was made according to the observations of cohorts that received sub-optimal regimens (too few doses) of otherwise protective vaccines during the field trials. Whereas significant efficacy was observed during years 1 and 2 of a Ty21a vaccine efficacy trial in Area Norte, Santiago, little or no residual immunity was observed during the 3–5 year follow-up [20].

Shifts in age-specific incidence during the vaccine trials offer insight into the true duration of protection from Ty21a vaccine, as maximum follow-up times were 7 years, 5 years and 3 years, with most trials still indicating measurable protection at the last follow-up point. Thus, duration (D) of efficacy for full-dose vaccinees was left as a free parameter and included in model fitting.

**Fig 3:**
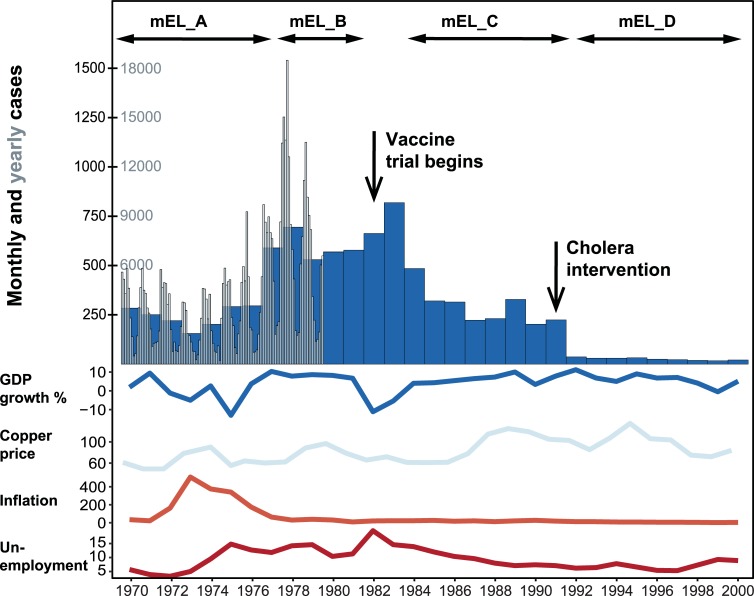
Typhoid fever and economic indicators over time in Santiago. Monthly and yearly incidence of typhoid, plotted with economic indicators including % change in GDP compared to 1960 as a baseline, price of copper in United States dollars per pound, inflation indicated by the annual change in the consumer price index, and unemployment percentage of the estimated labor force [45]. Timing of the interventions and mEL parameters are depicted.

Longitudinal trends in the pre-vaccine era indicate a relatively stable incidence from 1970 to 1976, with notable increases in 1977–1978 and 1982–1983 (Fig 3). Apparent shifts in GDP growth and copper prices during these years may be loosely correlated with changes in irrigation practices or food purchase habits (Fig 3) [45], and may therefore drive exposure to the long-cycle CCVT. Our model included multipliers on the long-cycle CCVT exposure frequency (mEL), for these pre-defined time periods plotted in Fig 3. We also explored whether the increase in cases from 1982 to 1983, beginning not long after initiation of the TFCP, could be attributed to increased diagnostics, by fitting a higher pA value for the years after 1982.

The multiplier mEL was assumed to be zero from 1992 to 2000, when exposure through the long-cycle was interrupted due to a prohibition of irrigation with sewage. Thus, the model was constrained exclusively to short-cycle transmission during this time (Table 2). Longitudinal changes in exposure to long-cycle CCVT may also have occurred during the vaccine period. After fitting vaccine duration parameters, a linear multiplier on long-cycle CCVT exposure frequency (mEL) was fitted between 1984 and 1992 to capture additional incidence reduction seen during the vaccine period that wasn’t captured by the vaccine.

### Model fitting

Parameters that were not identified from the literature were estimated through model fitting. The estimated parameter values were identified from pre-defined ranges (Table 1) using a gradient ascent algorithm, which iteratively maximizes a combined log-likelihood to approach a local optimum, calculated from the fit of the model to identified data components (S1 Appendix). The TFCP and CMoH collected and reported data from many independent sources, leading to heterogeneity in years of reporting, age tranches and timeframes of each data component. Data were collated from the authors’ personal files (S1 dataset), with additional components and age tranches shared from recent exploratory initiatives [46]. Data from simulated years were extracted to match to report years for calculation of each component of the likelihood.

Data related to typhoid incidence was informed by passive surveillance, meaning the patient or the parent/caretaker of a sick child had to make the decision to actively seek health care. The surveillance activities and vaccine field trials were carried out in parts of Santiago and in an era when the vast majority of the population (except the very wealthy) sought acute care at health centers (consultorios) run by the government. Typhoid fever was a notifiable disease, meaning all healthcare providers were required to report diagnosed cases. Infections reported to the TFCP and CMoH were assumed to be represented by the acute infection disease state in the model (Fig 1).

Four distinct components of the data were used in the first stage of model fitting (S1 Table), which estimated all free parameters in the model with the exception of mEL_C (Table 1). Age distribution before, during and after the vaccination period (1971–1992) and pre-vaccine period seasonality (1970–1979) were derived from Ministry of Health reports. Age distribution of incidence for the year 1984 was excluded due to missing age-specific demographic data from that year. Chronic carrier prevalence (1980) was obtained from literature estimates, which multiply population cholelithiasis prevalence by gallbladder carriage of *S*. Typhi. Annual incidence in the pre-vaccine period (1970–1983) and post environmental intervention (1993–1996) were derived from Ministry of Health reports, and used for the first stage of model fitting.

Annual incidence during the vaccination period was withheld in the first stage of model fitting, due to the potential for the estimate of the free parameter for vaccine duration (D) to be influenced by the changing incidence rates during the vaccination period. Due to our assumptions of long-lasting immunity after repetitive exposures and infections in endemic locations, we similarly assume that the age distribution of typhoid in the model is robust to incidence rate changes in the short term, and therefore attribute changes to age distribution during the vaccine period to be a result of the vaccine.

The second stage of model fitting involved fixing the parameters estimated in the first stage, and utilizing annual incidence data from the vaccine period (1983–1992) to fit mEL_C. This allowed us to independently estimate exposure-related changes during the vaccine period, with vaccine duration and other individual-level parameters fixed.

## Results

**Fig 4:**
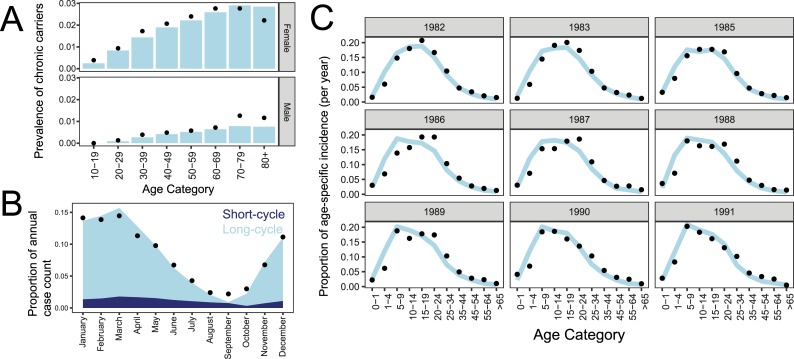
Fit of model to Santiago dynamics of typhoid fever. Fit of model (in blue) to data (•) informing the prevalence of chronic carriers(A), typhoid seasonality, divided by cases resulting from exposure to the short- vs. long cycle (B), and the distribution of age-specific typhoid incidence during and after the vaccine period (C).

**Fig 5:**
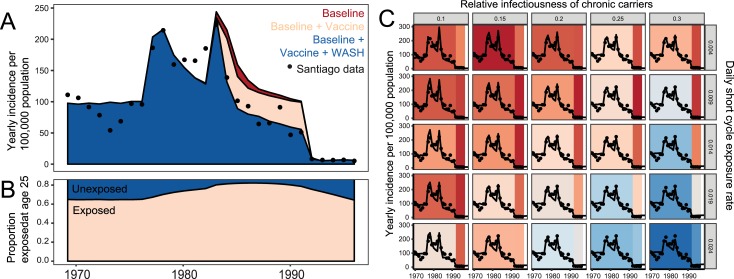
Time trends in the model and data. A. The model’s best fit estimates for vaccine and environmental impacts during the study period. B. Percentage of 25 year-olds that have ever been exposed to *S*. Typhi over the study period, as predicted by the model. C. Longitudinal model estimates with Santiago data (•) for a range of ES and Rc values. Background color indicates scaled likelihood values for the model’s fit to pre-1976 and post-1992 Santiago data, with red indicating better fits.

Fit of the model to Santiago dynamics of age distribution, typhoid seasonality, and prevalence of chronic carriers is shown in Fig 4. Fits of the model to longitudinal trends in reported typhoid fever are plotted in Fig 5A, with estimated parameter values summarized in Table 1. Increases in exposure to the long cycle CCVT in simulated years 1978 and 1983 resulted in an increased estimated population immunity (measured by percentage of the simulated population exposed at age 25) prior to the vaccination period (Fig 5B), which is a likely contributor to the decline in incidence in the following years. We also modeled a scenario where the increase in cases in 1983 was due to improved diagnostics, which led to a poor fit of the model to data for years after 1983 (S1 Fig).

The model estimates that the majority of typhoid infections result from long-cycle transmission in the endemic period but the ratio to short-cycle infections varies seasonally (Fig 4B). Parameters driving seasonality estimate an asymmetrical exposure, with a short ramp-up beginning on day 279 (October 6^th^), a duration of peak long-cycle exposure of 108 days ending on January 22^nd^, and a gradual ramp down of 227 days. Predictions from Chile in 1979 based on temperature estimate the growing season to begin August 30^th^ [47], somewhat preceding our estimated exposure start.

Population immunity drives the adult age distribution of simulated typhoid fever in Santiago, which is created by both immunity after clinical typhoid and a high incidence of immunity-boosting repetitive sub-clinical infections. The best-fit model estimates a protection per-infection parameter (P) of 99.8%, indicating that each initial acute clinical or subclinical *S*. Typhi infection causes a substantial reduction in susceptibility to subsequent clinical typhoid in the model. Repeat episodes of typhoid fever have been observed in individuals who participated in experimental human challenge/re-challenge studies [48], or who were members of circumscribed populations that experienced successive typhoid epidemics [49]: these data indicate only modest protection against subsequent typhoid conferred by the initial clinical infection. It is presumed that recurring subsequent exposures in hyper-endemic areas repetitively boost immunity and maintain long-lived protection [50]. No recent studies have addressed clinical reinfections in endemic settings. The best-fit model estimates a large proportion of sub-clinical infections, with the symptomatic fraction of typhoid infection (pA) at 5.3%.

When fitting the model to the estimated chronic carrier prevalence in Santiago, the best-fit probability of carriage following acute or subclinical infection of persons with gallstones (pC) was 10.8%. When multiplied by the gallstone carriage prevalence rates [15], we independently estimate the probability of carriage due to infection at age-specific rates that are lower than pre-antibiotic era estimates from New York State, with an age-adjusted rate of 1.5 vs. 2.9% (Table 4, S1 Table) [39].

**Table 4:** Comparison of typhoid chronic carriage rates in literature vs. model predictions.

Age bin	Gender	Typhoid cases in New York State, 1930–1939 *	Predicted number of resulting chronic carriers based on model parameters	Observed chronic carriers *
<10	Female	281	0	0
10–19		411	4.31	1
20–29		238	6.01	5
30–39		193	8.98	12
40–49		122	6.81	20
50–59		78	5.05	9
60–69		21.33	1.59	2
70–79		21.33	1.59	2
80+		21.33	1.28	2
<10	Male	347	0	2
10–19		491	0	2
20–29		341	1.66	7
30–39		216	3.13	6
40–49		173	3.12	6
50–59		110	2.35	10
60–69		21.67	0.58	1.33
70–79		21.67	1.02	1.33
80+		21.67	0.94	1.33
**Total**		**3130**	**48.43**	**90**
**Carrier probability**			**0.015**	**0.029**

Columns marked with * are derived from Ames and Robins, 1943 [39]. In the absence of further resolution of the 60+ age bin in Ames and Robins, we assume a uniform distribution across the 60+ age tranches listed above.

During the period of hyper-endemic transmission in the Santiago model, one could offset low levels of short-cycle transmission (ES) with high levels of chronic carrier infectiousness (rC) to capture longitudinal trends, based on likelihood values (Fig 5C). The availability of incidence data after the interruption of long-cycle transmission in 1991 allowed us to constrain these two parameters that would otherwise be unidentifiable. This is additionally aided by our prevalence estimate of chronic carriers, which is unknown in most endemic locations. The model estimated the relative infectiousness of chronic carriers to be 24% of the infectiousness of acute cases and a short-cycle transmission rate of 0.0093.

Other parameters were less identifiable. Specifically, a trade-off exists between acute infectiousness (AI) and exposure to the long-cycle (EL), the dominant route of transmission in this context. As acute infectiousness is the primary driver of infectious dose in the model, we can simulate high or low-dose scenarios by adjusting values for acute infectiousness. A higher value of long-cycle exposure frequency (EL) can offset a lower value of dose, and vice-versa, resulting in a stable probability of infection despite fluctuating values. Fitted values of 13,436 CFU and 0.54 were estimated for AI and EL, respectively (Table 1), but further studies to help identify one or both of these parameters would be valuable.

The best-fit model estimates duration of the efficacy of Ty21a vaccine to be 8.4 years, as determined by the model’s fit to age distributions during and after the vaccination period (Fig 4C). The model estimates that in addition to the vaccination-related decline beginning in 1983, there was an estimated 23–53% reduction in exposure to the long-cycle over this period, increasing linearly until after 1991 (Fig 5A).

We estimated vaccine impact by comparing simulations of WASH-only and WASH+vaccine scenarios, using best-fit parameters. We see a maximum estimate of 11.7% reduction of cases across all age groups in the year 1985. With 5.3% of the overall population receiving full dose vaccines by the end of the trial, and an additional 4.6% receiving partially protective formulations, this indicates indirect protection of non-vaccinated age groups is likely occurring. The high coverage and direct protection within vaccinated age groups is reflected in the shifts in age distribution of incidence between 1982 and 1991, with peak age-specific incidence shifting from 15–19 years of age in 1982, to 5–9 years of age by 1991 (Fig 4C).

## Discussion

Between 1979 and 1993, through a multi-faceted applied public health research agenda, the Chilean TFCP generated data on the magnitude of the human chronic carrier reservoir, modes of transmission and impact of vaccine and sanitation interventions in Santiago [13]. We utilized these data in a mathematical model to understand the mechanisms of transmission in this setting, and to estimate the impact of both vaccine and environmental control measures.

Similar to observations in many current typhoid-endemic locations [1], the age distribution of typhoid in Santiago has a paucity of adult cases relative to children. Two primary mechanisms can create this pattern in the mathematical model: i) the degree of immunity after infection; ii) and the incidence rate of clinical and sub-clinical infections. Both mechanisms appear to play a role in Santiago. Parameter estimates when fitting the mathematical model to the age distribution of typhoid in Santiago suggest robust immunity after clinical and sub-clinical infection, with a very low probability of repeated infection after an initial infection. Our model-estimated immunity after infection is much higher than what has been demonstrated in challenge studies [48], but studies of repeat infections in endemic settings are lacking. Additionally, the model estimates the occurrence of approximately 19 sub-clinical infections for each clinical case, leading to a large amount of circulating *S*. Typhi infection that remains ‘unreported’ in the model.

Population-based seroprevalence surveys that detect long-lived anti-flagella H:d responses from both prior sub-clinical and clinical typhoid infections cumulatively over time offer insight into the levels of circulating disease that may go undetected by clinical surveillance [13,50]. A cross-sectional prevalence survey of *S*. Typhi H antibody in Santiago in 1978 found that approximately 50% of 25 year-olds had a reciprocal titer ≥ 40. Estimates from our model (Fig 5B) corroborate the estimate derived by seroepidemiology and predict that 60–70% of the population has been infected by the age of 25 during the pre-vaccination period. Absent data informing decay rates of the H antibody over time, the higher percentage of estimated individuals ever having been exposed compared to antibody prevalence may be explained by the decay of H antibody over time, and at a minimum supports our finding that many subclinical or mild infections occur for each reported clinical case [13].

The proportion of incident typhoid cases reported to public health authorities is notoriously variable among modern typhoid-endemic healthcare locations and can be attributed to differences in treatment-seeking, volumes of blood drawn for culture, and microbiological methods. If we assume the estimated parameter specifying immunity after initial infection is consistent across diverse locations, differences in the adult age distribution of typhoid should only be driven by the rate of disease transmission. The shape of the adult age-specific case distribution may better indicate the force of infection than incidence rate based on an unknown case reporting fraction [13,50]. Serological surveillance is needed to confirm these observations in modern typhoid-endemic locations.

We estimated a probability of carriage after infection that is lower than the age-specific rates estimated in the pre-antibiotic era (Table 4) [39]. This is expected due to the ability of certain antibiotics (fluoroquinolones, e.g., ciprofloxacin) to diminish chronic carriage after treatment of acute infection. Indeed, a longer (4-week) course of these antibiotics can even eliminate established chronic gallbladder carriage without cholecystectomy [51,52]. Our estimate was dependent on the accepted dogma that sub-clinical cases can lead to chronic carriage, while Ames and Robins only followed-up clinically detected cases [39].

We utilized two pieces of data, the prevalence of chronic carriers and the incidence after the environmental intervention, to estimate parameters that typically are unidentifiable: the infectiousness of chronic carriers and the short-cycle transmission rate. When investigating persistence after extreme WASH interventions in modern endemic locations, one should consider that the Santiago results are likely a lower-bound for short-cycle transmission rates, due to the widespread availability of potable water in Santiago households and other improved WASH indicators. Because we see sustained but progressively diminishing transmission in Santiago after interruption of long-cycle transmission in 1991, we posit that chronic carriers transmitting through the short-cycle are largely responsible. The contribution of carriers should be studied intensively in future projects aimed to achieve accelerated control (and eventually local elimination) of typhoid, once amplified long-cycle endemic transmission has been curtailed, including after widespread vaccination with effective vaccines that alter the susceptibility of the population.

The impact of large-scale use of Ty21a vaccine was assessed within the context of other potential changes occurring during the 1980s in Santiago. Pre-vaccination incidence increases during 1977 and 1982 led to a subsequent decrease of naïve individuals in the model, leading to an estimated decline in incidence over time independent of vaccination (Fig 5A). Shifts in the age distribution of typhoid fever incidence during the vaccination period were valuable for understanding the duration of efficacy of Ty21a, which the model estimates to be ~1.5 years longer than the maximum duration of protection documented in field trials (8.4 versus 7 years). Data additionally support longer durations of efficacy for some less protective formulations and immunization regimens of Ty21a vaccine, past the follow-up times published in literature [19]. For example, over six years of follow-up, three doses of enteric coated capsule and gelatin capsule formulations given in long intervals (21 days) between doses exhibited 55.1% efficacy (95% CI, 38.2–67.4) and 35.0% efficacy (95% CI, 13.7–51.0), respectively, while the enteric-coated capsule formulation administered at short interval (2 days) conferred 62.7% (95% CI, 47.7–73.5) efficacy (S2 Table). When evaluating vaccination impacts on a population level, it will be important to consider potential shifts in population immunity and age distribution of infection, in the case of an age-targeted vaccine.

The model shows that with Ty21a use typhoid incidence at the population level falls more than expected based on direct protection only, indicating indirect effects of the vaccine, which have been described using other methods of analysis [24]. Our finding depends on modeling reduced shedding in protected vaccinees, an assumption documented in Ty21a challenge studies [53]. Additionally, this finding is influenced by the model structure, which assumes a well-mixed environmental reservoir, consistent with the known transmission route. In locations without known transmission routes, the assumption of a well-mixed pool of infection may not be valid and may over-estimate indirect effects. Investigations into spatial scales of transmission are needed when modeling modern endemic locations.

Utilizing the model’s trade-offs between dose-response and exposure frequency, driven by parameters acute infectiousness (AI) and long-cycle exposure frequency (EL), typhoid dynamics can be simulated at both high-dose and low-dose scenarios. The unidentifiability of these parameters is a limitation of model structure, at present, and studies quantifying estimated exposure levels and frequency would greatly improve our understanding of transmission dynamics and vaccine efficacy in relation to infectious dose.

The vaccine efficacy estimates we used in the model were derived from field trial data, which do not account for potential differences in vaccine efficacy in relation to variations in the size of the inoculum ingested. Thus, our impact estimates did not account for potential variation in infectious dose. Experimental challenge studies that assessed the efficacy of parenteral killed whole-cell typhoid vaccines in volunteers showed that high inocula could overwhelm the protective effect of vaccines efficacious against lower doses [34]. Since a similar concern was raised by investigators who assessed the efficacy of a Vi conjugate vaccine in a challenge model [54], this should be considered when projecting the impact of new conjugate vaccines.

In summary, this study utilized unique datasets collected during multiple stages of endemicity and control in Santiago, Chile. Paired with mathematical modeling, we aimed to better understand both the complex dynamics contributing to sustained transmission in this setting. Modeling also allowed us to estimate the contributions of mass use of vaccine, in a time when other water and sanitation measures were underway. Our findings support the use of typhoid vaccines to reduce transmission, but also highlight the importance of identifying and intervening upon the critical long-cycle transmission pathways, allowing for targeted and sustained control.

## Supporting information

S1 Appendix Parameter estimation. (DOCX)

S1 Table Derivation of age-specific chronic carriage rates. (DOCX)

S2 Table Results of six years of follow-up of a randomized, placebo-controlled field trial in Area Occidente, Santiago. (DOCX)

S1 Fig Additional modeling scenarios. Model output exploration of 1983 peak incidence due to improved diagnostic (A) or increase in incidence (B), with and without vaccine, fit to Santiago data (•).
(EPS)

S1 Dataset Data release. Data includes reports from the TFCP and CMoH collected before, during and after the vaccine trial, digitized by the authors.
(XLSX)
