# Supplementary material for: Typhoid fever in Santiago, Chile: Insights from a mathematical model utilizing venerable archived data from a successful disease control program
Source: PLoS Negl Trop Dis. 2018 Sep 6;12(9):e0006759. doi: 10.1371/journal.pntd.0006759 (PMC6143279; doi:10.1371/journal.pntd.0006759)
Supplement: S1 Appendix — (DOCX) [file pntd.0006759.s001.docx]

S1 Appendix: Parameter estimation

The fitted parameters used in our model were estimated by numerical maximization of a likelihood function, using a gradient ascent algorithm. The model fitting stages are outlined in Table A. The individual-level parameters were fixed in stage 2 to prevent changes in annual incidence over the vaccination period (1984-1992) from influencing the fit of individual-level parameters and vaccine duration. The beta-binomial distribution (1) was used to fit typhoid incidence and chronic carriage in the population, while the dirichlet multinomial distribution (2) was used to age distribution of incidence and seasonality. The log of each likelihood component was combined into a single log-likelihood using weights (3), which were used to upweight age distributions in the post-vaccination period, and to prioritize fitting of the two peaks in incidence, which, as outlined in the primary text, were assumed to be true changes in incidence.

**Beta-binomial:**

$$L=\prod_{j=1}^{J} \frac{N!}{\zeta_{j}!(N -\zeta_{j})!} \frac{\Gamma(2+N)\Gamma(1+I_{j} +\zeta_{j})\Gamma(1+2N -I_{j} -\zeta_{j})}{\Gamma(1+I_{j})\Gamma(1+N -I_{j})\Gamma(2+2N)} (1)$$

J= number of observation time points

I_j_= number of events (i.e. disease events, number of chronic carriers) simulated at year j

N= total simulated population

ζ_j_ = rate of event at year j

**Dirichlet multinomial:**

$L=\frac{N!}{{\prod_{k=1}^{K} x_{k}!}} \frac{\Gamma(A)}{\Gamma(A+N)}\prod_{k=1}^{K} \frac{\Gamma(\alpha_{k}+ x_{k})}{\Gamma(\alpha_{k})}$ (2)

K = number of categories (i.e. the number of age bins or months in year)

A = total observed cases in Santiago over time period

α_k_ = observed cases in Santiago in category k

N = total observed cases in simulation over time period

x_k_ = observations in simulation in category k

**Combined likelihood function:**

$L=exp[ \sum_{i=1}^{C} \alpha_{i}*\log\left( L_{i} \right)]$ (3)

C= number of components

α_i_= component weight

L_i_= component likelihood

|  | **Stage 1** | | |  | **Stage 2** | | |
| --- | --- | --- | --- | --- | --- | --- | --- |
| **Likelihood components** | **Component** | **Years** | **Likelihood function** | **Weight** | Annual incidence | 1983-1992 | Beta-binomial |
|  | Age distribution | 1971-1992 | Dirichlet multinomial | 1, 5 for years 1990- 1992 |  |  |  |
|  | Seasonality | 1970-1979 | Dirichlet multinomial | 1 |  |  |  |
|  | Annual incidence | 1970-1983, 1993-1996 | Beta-binomial | 1, 5 for years 1983, 1978 |  |  |  |
|  | Chronic carrier prevalence | 1980 | Beta-binomial | 1 |  |  |  |
| **Free parameters** | AI, P, EPS, PD, RDD, RUD, ES, EL, pC, pA, Rc, S, mEL_A, mEL_B, D | | |  | mEL_C | | |
| **Fixed parameters** | Table 2 parameters | | |  | Table 2 parameters, AI, P, EPS, PD, RDD, RUD, ES, EL, pC, pA, Rc, S, mEL_A, mEL_B, D | | |

**Table A. Outline of model fitting stages with components, years, and weights.**
