# Supplementary material for: Typhoid fever in Santiago, Chile: Insights from a mathematical model utilizing venerable archived data from a successful disease control program
Source: PLoS Negl Trop Dis. 2018 Sep 6;12(9):e0006759. doi: 10.1371/journal.pntd.0006759 (PMC6143279; doi:10.1371/journal.pntd.0006759)
Supplement: S1 Table — (DOCX) [file pntd.0006759.s002.docx]

| **Age bin** | **Gender** | **Gallstone prevalence*** | **Probability of carriage given gallstones (pC)** | **Carrier probability** |
| --- | --- | --- | --- | --- |
| <10 | Female | 0 | 0.108 | 0 |
| 10-19 |  | 0.097 | 0.108 | 0.010 |
| 20-29 |  | 0.234 | 0.108 | 0.025 |
| 30-39 |  | 0.431 | 0.108 | 0.047 |
| 40-49 |  | 0.517 | 0.108 | 0.056 |
| 50-59 |  | 0.600 | 0.108 | 0.065 |
| 60-69 |  | 0.692 | 0.108 | 0.075 |
| 70-79 |  | 0.692 | 0.108 | 0.075 |
| 80+ |  | 0.555 | 0.108 | 0.060 |
| <10 | Male | 0 | 0.108 | 0 |
| 10-19 |  | 0 | 0.108 | 0 |
| 20-29 |  | 0.045 | 0.108 | 0.005 |
| 30-39 |  | 0.134 | 0.108 | 0.014 |
| 40-49 |  | 0.167 | 0.108 | 0.018 |
| 50-59 |  | 0.198 | 0.108 | 0.021 |
| 60-69 |  | 0.247 | 0.108 | 0.027 |
| 70-79 |  | 0.435 | 0.108 | 0.047 |
| 80+ |  | 0.4 | 0.108 | 0.043 |

**S1 Table. Derivation of age-specific chronic carriage rates**

We aimed to compare the observed rate of carriage after clinical infection observed in Ames and Robins, with what would be expected based on our Santiago model assumptions and fitted parameters (Table 4). We multiplied prevalence of gallstones measured in Santiago [15] by the probability of carriage given gallstones estimated in model fitting, pC, (Table 1) to calculate an overall probability of carriage after infection.

Values marked with * are derived from Levine, 1982 [15]
