# Supplementary material for: Typhoid fever in Santiago, Chile: Insights from a mathematical model utilizing venerable archived data from a successful disease control program
Source: PLoS Negl Trop Dis. 2018 Sep 6;12(9):e0006759. doi: 10.1371/journal.pntd.0006759 (PMC6143279; doi:10.1371/journal.pntd.0006759)
Supplement: S2 Table — (DOCX) [file pntd.0006759.s003.docx]

| Sept 1983 - Aug 1989 | Enteric coated capsule, long | Enteric coated capsules, short | Gelatin capsules, long | Gelatin capsules, short | Placebo |
| --- | --- | --- | --- | --- | --- |
| No. of schoolchildren | 21,598 | 22,170 | 21,541 | 22,379 | 21,904 |
| No. of confirmed  typhoid fever cases | 54 | 46 | 78 | 111 | 122 |
| Incidence/10^5^/72 months | 250.0 | 207.5 | 362.1 | 496 | 557.0 |
| Efficacy  (95% CI) | 55.1%  (38.2 – 67.4) | 62.8%  (47.7 – 73.5) | 35.0%  (13.7 – 51.0) | 11.0%  (15.1 – 31.1) | - |

**S2 Table. Results of six years of follow-up of a randomized, placebo-controlled field trial in Area Occidente, Santiago.** Comparison of the efficacy of three doses of Ty21a live oral typhoid vaccine administered to Chilean schoolchildren in two different formulations and two different spacing between doses.

One previous publication reported results of the first three years of follow-up of these randomly allocated groups in the Area Occidente field trial [1]. Another prior publication reported evidence that the efficacy of the enteric-coated capsule formulation administered at a 2-day interval between doses extended through seven years of follow-up [2].

Enteric-coated capsules (that had been treated with hydroxyprophymethylcellulosephthalate) contained lyophilized vaccine. They resisted opening in the presence of gastric acid but opened upon arrival in the duodenum where the pH was > 6.5 [1].

Each dose of the gelatin capsule formulation consisted of one gelatin capsule containing lyophilized vaccine and two additional gelatin capsules each containing 0.5 gm of NaHCO_3_ [1].

Long interval was 21 days between doses. Short interval was two days between doses.

Details of the design and methods of the Area Occidente field trial have been previously reported [1].
